# How Television Fast Food Marketing Aimed at Children Compares with Adult Advertisements

**DOI:** 10.1371/journal.pone.0072479

**Published:** 2013-08-28

**Authors:** Amy M. Bernhardt, Cara Wilking, Anna M. Adachi-Mejia, Elaina Bergamini, Jill Marijnissen, James D. Sargent

**Affiliations:** 1 Norris Cotton Cancer Center, Geisel School of Medicine at Dartmouth, Lebanon, New Hampshire, United States of America; 2 Department of Pediatrics, Geisel School of Medicine at Dartmouth, Lebanon, New Hampshire, United States of America; 3 Public Health Advocacy Institute, Northeastern University School of Law, Boston, Massachusetts, United States of America; 4 Developmental Psychopathology, Behavioural Science Institute, Radboud University, Nijmegen, The Netherlands; University of Missouri-Kansas City, United States of America

## Abstract

**Objectives:**

Quick service restaurant (QSR) television advertisements for children’s meals were compared with adult advertisements from the same companies to assess whether self-regulatory pledges for food advertisements to children had been implemented.

**Methods:**

All nationally televised advertisements for the top 25 US QSR restaurants from July 1, 2009 to June 30, 2010 were obtained and viewed to identify those advertising meals for children and these advertisements were compared with adult advertisements from the same companies. Content coding included visual and audio assessment of branding, toy premiums, movie tie-ins, and depictions of food. For image size comparisons, the diagonal length of the advertisement was compared with the diagonal length of salient food and drink images.

**Results:**

Almost all of the 92 QSR children’s meal advertisements that aired during the study period were attributable to McDonald’s (70%) or Burger King (29%); 79% of 25,000 television placements aired on just four channels (Cartoon Network, Nickelodeon, Disney XD, and Nicktoons). Visual branding was more common in children’s advertisements vs. adult advertisements, with food packaging present in 88% vs. 23%, and street view of the QSR restaurant present in 41% vs. 12%. Toy premiums or giveaways were present in 69% vs. 1%, and movie tie-ins present in 55% vs. 14% of children’s vs. adult advertisements. Median food image diagonal length was 20% of the advertisement diagonal for children’s and 45% for adult advertisements. The audio script for children’s advertisements emphasized giveaways and movie tie-ins whereas adult advertisements emphasized food taste, price and portion size.

**Conclusions:**

Children’s QSR advertisements emphasized toy giveaways and movie tie-ins rather than food products. Self-regulatory pledges to focus on actual food products instead of toy premiums were not supported by this analysis.

## Introduction

Global food companies have an influential impact on public health, [1] and the enormous resources they direct toward marketing and branding of unhealthy foods [2] has generated scrutiny of how food is marketed to children. [3] Fast food consumed by children away from home in quick service restaurants (QSRs) is of particular concern, as it is linked to increased calorie intake [4] and decreased diet quality. [5] Further, increases in the proportion of calories consumed away from home corresponds with the onset of widespread obesity in the population. [5] Finally, higher consumption of fast food has been linked with larger increases in body mass index over time [6].

Fast food intake in children may be influenced by fast food marketing. [7] Televised food commercials played on children’s television channels often advertise nonessential foods and frequently contain premiums and promotional characters. [8] In 2006, U.S. QSR chains reported to the Federal Trade Commission (FTC) that they spent $161 million marketing to 2–11 year olds (56% on television advertising). Of the money QSR chains spent, $74.4 million (46%) went to cross-promotions to tie their meals to movies, television shows, and animated characters. [9] An additional estimated $360 million was spent on the toy premiums themselves. [10] Exposure to food advertisements has been shown to alter eating choices and behaviors, [11] and associating food with animated characters enhances a child’s perceived food taste and preference. [9], [12] Obese children may be highly susceptible to food advertising. [13], [14] Fast food advertising exposure is associated with higher fast food consumption in children, [15] and fast food branding has been shown to influence taste preferences [16].

In the United States, food composition, ingredient labeling and health claims are subject to federal regulation by the Food and Drug Administration (FDA). General advertising or marketing of food is primarily regulated by the Federal Trade Commission (FTC). [17] (For a description of the regulatory role on food advertising for each agency, see http://www.ftc.gov/bcp/policystmt/ad-food.shtm). At the state level, state attorneys general have broad powers to regulate the food industry. Food marketing must not be false, deceptive or unfair under applicable federal and state laws.

A self-regulatory system also exists, run by the Better Business Bureau (BBB) and funded by private industry. Since 1974 the BBB has operated the Children’s Advertising Review Unit (CARU), which maintains a set of marketing guidelines focused on tactics used to market foods and other products to children. For example, CARU’s Guidelines state that: “[a]dvertisers should recognize that their use of [toy] premiums... has the potential to enhance the appeal of their products to children” and that “since children have difficulty distinguishing product from premium, advertising that contains a premium message should focus the child’s attention primarily on the product and make the premium message clearly secondary.” [18] Since 2006, the BBB also has run the Children’s Food and Beverage Advertising Initiative whereby companies pledge that food advertising to children will feature only foods and meal combinations that meet certain nutrition criteria. [19] The combination of CARU’s toy premium guideline and CFBAI’s emphasis on the nutritional value of the foods advertised to children suggest that food should be the focus of children’s QSR advertising.

In a 2006 study conducted prior to the CFBAI, Connor [20] assessed food marketing on four hour blocks of children’s programming and found fast food advertisements aimed at young children (from McDonald’s, Chuck E Cheese’s, and Wendy’s) promoted branding. Branding seeks to create positive associations with a company or product and is achieved through images of children engaged in fun or exciting activities, mascot imagery and licensed characters as opposed to the actual characteristics of the product itself, in this case food. We examined all television advertisements from the 25 top US QSR restaurant chains that aired on national television. We wished to determine the proportion of QSR companies that ran any television advertisements aimed at children during the study period, to determine if this practice was common for QSR companies. Secondly, for companies that ran advertisements aimed at children, we contrasted QSR television marketing approaches to children versus adults, conducting a content analysis of the children’s advertisements and a matched set of adult advertisements from the same companies. We aimed to determine if companies were adhering to the CARU guidelines that mandated a focus on food, rather than premiums and tie-ins, in children’s QSR advertising. A continued focus in children’s advertisements on nonfood items, particularly if advertisements aimed at adults emphasized food, would imply that the self-regulatory guidelines are ineffective.

## Methods

We obtained the television advertisements for the top 25 quick service restaurants for 2008 named in Quick Service Magazine and based on 2008 system-wide sales in the United States. Advertisements were purchased from an ad agency which monitors all cable and network television. We identified any QSR television ad placement that aired during a 1 year period (July 1, 2009 to June 30, 2010) on national television (N = 1135). All advertisements were reviewed to determine if the product being marketed was a “children’s meal” specifically packaged for children (e.g., the McDonald’s Happy Meal). Unique advertisements were identified by content and length. Some of the 30-second children’s advertisements contained two distinctly themed 15-second segments back-to-back. Despite the incongruent content, these advertisements were treated as one unique 30-second ad because that is how they were aired.

Each children’s advertisement was matched for length with an ad aimed at adolescents/adults that was randomly selected from the adult ad pool from that company. All of the advertisements were evaluated by 2 coders and all discrepancies on all ad content were resolved by a third person. We coded the advertisements through visual assessment and audio track transcripts. We viewed every 16th frame, with each frame visually assessed for the presence of variables indicative of branding(visual depiction of logo, mascot, food packaging, street view of the restaurant), the presence of giveaways (toys) and cross-promotions, and food products (food, drinks, and healthy food (apples or milk)). For each frame where food or drink was clearly recognizable, the largest food image was assessed for size, which was measured along its longest dimension and compared with the diagonal of the ad frame. For images that contained multiple food items, the diagonal of the entire composite image was measured.

A written audio track transcript for each QSR food ad was created and a total word count established for each ad. Coding rules were established to associate words from the transcript with each variable of interest, including those listed above as well as any reference to the food itself (spicy, freshly baked, 100% pure beef), portion size (big, third pounder, double) or price (value, dollar, “a steal”). For words with multiple meanings, coders were instructed to associate them with one variable of interest according to a pre-determined hierarchy.

### Statistical Analysis

We first compared the proportion of advertisements having any visual depiction of a variable of interest using the chi square test. Among advertisements containing one or more frames for the variable of interest, a two-tailed t-test of means was performed to assess whether adult advertisements had, on average, a higher or lower percentage of frames with the variable depicted, compared with the children’s advertisements. Food and drink size was assessed as a percent of the screen (i.e., the advertisement) diagonal. For frames with food and drink, percent of the screen diagonal was regressed as a function of adult vs. children’s ad while controlling for vendor and accounting for clustering of the data at the ad-level. Word counts for the advertisement audiotrack were also assessed. Adult advertisements had significantly more words in the audio script compared with children’s advertisements (mean 58 vs. 39, respectively), so words associated with a variable were assessed as a percentage of the total word count. All percentages were compared using the t-test, with a p-value of <0.05 considered statistically significant.

## Results

### Which Quick Service Restaurant Chains Market to Children on Television?

The top 25 QSR restaurants covered in the study are listed in Table 1, along with 2009 revenues, whether any advertising involving childrens’ meals was identified from our sample of 1135 national television advertisements, and the number of unique children’s advertisements that aired during the study period. Only 3 QSR companies were found to advertise to children; those products were Burger King Kids Meals, McDonald’s Happy Meals, and Subway Fresh Fit for Kids. During the study observation period, there were 62 unique McDonald’s Happy Meal and 30 Burger King Kid’s Meal advertisements, and only 3 from Subway. The sample from Subway was too small to include in any subgroup analyses by company, and these advertisements were dropped from the dataset. This left 92 children’s advertisements from McDonald’s and Burger King and 92 matched adult McDonald’s and Burger King advertisements for the analysis. Data were available for where and when the advertisements were placed on national television for 180 of the 184 advertisements.

**Table 1:** National televsion adertising aimed at children, top 25 QSR restaurants in 2009, United States.

Top 25 Fast FoodRestaurant Chains	2009 U.S. Revenues(millions of dollars)	Children’s Meal Television Marketingon National Television, 2009–10	Number of UniqueChildren’s Meal Ads*
McDonald’s	31	YES	62
Subway	10	YES	3
Burger King	9	YES	30
Starbuck’s Coffee	8.4	NO	0
Wendy’s	8.3	NO	0
Taco Bell	6.8	NO	0
Dunkin’ Donuts	5.7	NO	0
Pizza Hut	5	NO	0
KFC	4.9	NO	0
Sonic	3.8	NO	0
Arby’s	3.2	NO	0
Jack in The Box	3	NO	0
Domino’s	3	NO	0
Chick - fil-A	3.2	NO	0
Panera Bread	2.8	NO	0
Dairy Queen	2.6	NO	0
Papa John’s	2.1	NO	0
Hardee’s	1.7	NO	0
Quiznos Subs	1.8	NO	0
Popeyes	1.6	NO	0
Carl’s Jr.	1.4	NO	0
Chipotle	1.5	NO	0
Panda Express	1.3	NO	0
Wataburger	1.2	NO	0
Church’s Chicken	1	NO	0

* That aired nationally between July 1, 2009 and June 32, 2010.

Over the one-year period, 44,062 McDonalds and 37,210 Burger King advertisement placements were identified on national television channels. McDonald’s placed a stronger emphasis on the child market, with 40% of its placements aimed at young children compared with 21% for Burger King. Thus, during the study period, over two-thirds of all placements for children’s fast food advertisements were attributable to the McDonald’s QSR chain. Table 2 shows the 4 top stations for placement of the children’s advertisements, along with placements for the matched adult advertisements on those same channels. Seventy-nine percent of placements for children’s advertisements occurred on just four children’s television stations–Cartoon Network, Nickelodeon, Disney XD, and Nicktoons–with one-third of placements occurring on Cartoon Network. The adult advertisments were rarely placed on these stations, lending validation to their categorization into childrens’ and adult categories.

**Table 2:** Top four stations for placement for children’s ads, with placement for adult ads on the same channels.

	Number of Placements	
Station	Children’s Ads (percent of all children’s ad placements)	Adult Ads
Cartoon Network	8267 (32.3)	0
Nickelodeon	4671 (18.3)	9
Disney XD	4135 (16.2)	0
Nicktoons	3176 (12.4)	0

### How do Children’s Fast Food Advertisements Differ Visually from Adult Advertisements?

The 184 McDonald’s and Burger King advertisements contained 8,831 individual frames, about equally split between children’s and adult categories. Statistically significant comparisons are discussed below (all visual comparisons are shown in Table 3).

**Table 3:** Visual comparisons between children’s and adult ads.

Ad characteristic	Percent of ads Showing			Percent of frames (mean) among ads showing		
	Children’s	Adult	P	Children’s	Adult	P
Branding						
Logo	100	100	NS	33	23	<0.001
Mascot	15	13	NS	20	19	NS
Food packaging	88	23	<0.001	23	11	0.001
Restaurant street view	41	12	<0.001	24	19	NS
Giveaway or cross-promotion						
Toy giveaway	69	1	<0.001	34		
Movie	55	14	<0.001	61	44	0.05
Food emphasis						
Food present	96	84	0.008	26	34	0.02
Healthy food (milk or apples)	78	0	<0.001	18		
Drink present	89	60	<0.001	22	21	NS

All comparisons are frames =  = 3 vs all others (0–2 =  = 0, 3 =  = 1).

#### Branding

Logo depictions were present in all advertisements. The percentage of frames with logos was significantly higher in children’s (33%) compared with adult advertisements (23%). Mascots were not more common in children’s advertisements, but this obscures a difference by company. McDonald’s featured Ronald McDonald only in children’s advertisements (23% of the time) whereas Burger King’s “The King” appeared only in adult advertisements (40% of the time). Food packaging was present in 88% of children’s advertisements vs. 23% of adult advertisements. Among advertisements depicting any food packaging, the package was shown more frequently in children’s advertisements (23% vs. 11% of frames in adult advertisements). A street view of the restaurant was present more often in children’s advertisements (41%) than adult advertisments (12%).

#### Premiums and cross-promotions

Toy premiums were present in 69% of children’s advertisements vs. 1% of adult advertisments with 34% of frames in those advertisements containing a visual reference to toy premiums. References to movies or other cross-promotions were present in 55% of children’s vs. 14% of adult advertisements. None of the movies in children’s advertisements were rated as appropriate for general audiences (Rated G); the PG-13 category comprised 22% of movies cross-promoted to children by McDonald’s and 50% by Burger King (the rest were PG).

#### Food

**Figure 1:**
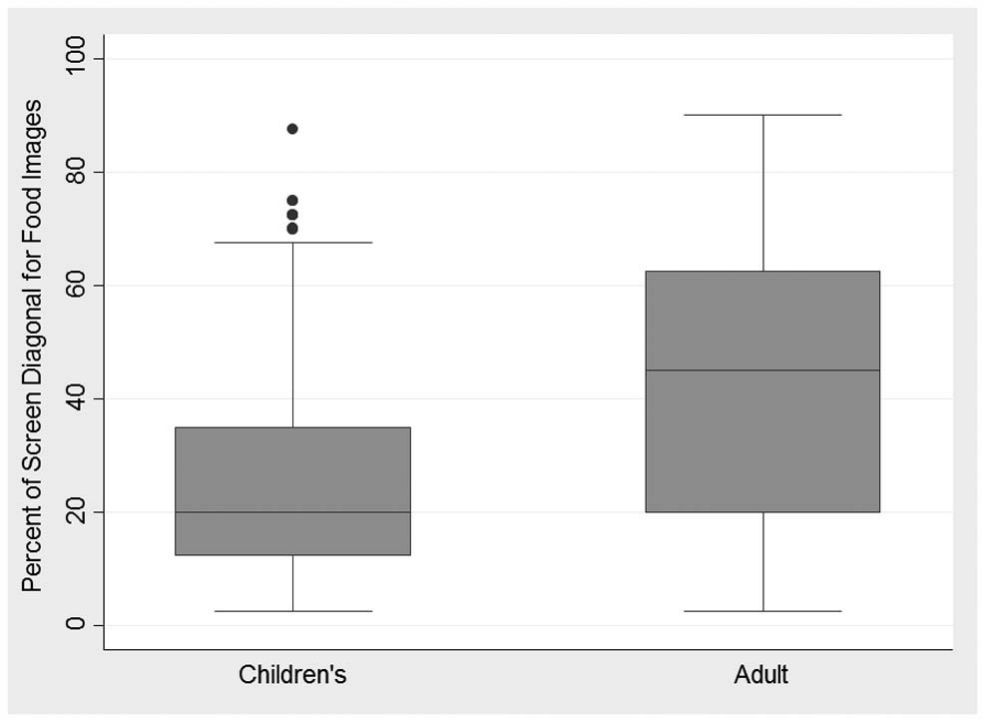
Distribution for the size of salient food images in television advertisements for Burger King and McDonald’s, by whether the advertisement was aimed at children or adults. Size is measured by the longest diagonal across the largest food image and is reported as the percentage of the screen diagonal. The top and bottom of each box represents interquartile range and the line in the middle of the box represents the median.

Food was present in almost all advertisements but drinks were present in a higher proportion of children’s advertisements. A higher proportion of adult ad frames (34%) showed food vs. children’s meal ad frames (26%). Healthy foods, milk and/or apple slices, were present in 78% of children’s advertisements and none of the adult advertisements and accounted for 18% of frames in the children’s meal advertisements. Figure 1 illustrates the significantly smaller size of food images in children’s vs. adult ads with box plots showing the median and interquartile range for food diagonal as a percentage of the screen diagonal in both types of ads. Median food image size was only 20 percent of the screen diagonal in children’s ads compared with 45 percent in adult ads. Drink images also represented a significantly smaller percentage of the screen diagonal for children’s compared to adult advertisements, but the differences were not as large as for food (median 15% vs. 20% respectively).

### How does the Audio Track of Children’s Advertisements Differ from Adult Advertisements?

**Figure 2:**
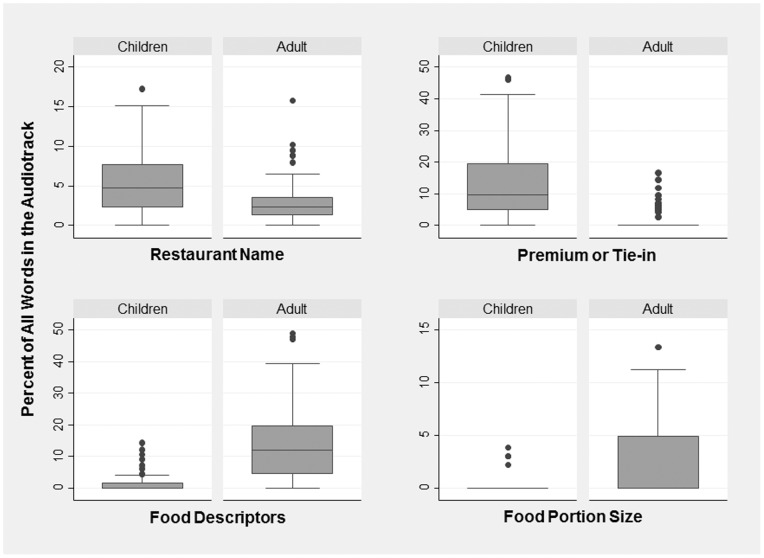
Percentage of all words in the audiotrack in advertisements for Burger King and McDonald’s that refer to restaurant name, premium or movie tie-in, food descriptors, or food portion size, by whether the advertisements were aimed at children or adults. The top and bottom of each box represents interquartile range and the line in the middle of the box represents the median.

Statistically significant comparisons for categorical word counts as a percentage of the total word count are illustrated in Figure 2, which shows box plots for the distribution for children’s vs. adult advertisements. Restaurant name occupied a median of 4.9% of the audio track for children’s advertisements and 2.8% for adult advertisements. Movie tie-ins or toys occupied a median of 12% vs. 1.5% of the audio script for children’s compared with adult advertisements. Food taste descriptors were much more common in adult compared with children’s audio tracks, occupying a median of 13.6% vs. 1.3%; the distribution for food descriptors in adult advertisements looked much the same as the distribution for descriptors for toy premiums or movie tie-ins in the children’s meal advertisements. Additionally, food portion size and price were both more common in adult compared to the children’s audio tracks–a median of 22.5% vs. 0.17% respectively for food portion size and 1.7% vs. 0.0% for food price (this comparison not illustrated in Figure 2).

## Discussion

This contemporary examination of QSR television advertising for the top 25 restaurants found that almost all children’s advertisements airing nationally in the U.S. were attributable to just two companies–McDonald’s and Burger King. These companies marketed children’s meals predominantly on four channels, including Nickelodeon which has been previously shown to primarily advertise foods of poor nutritional value. [21] Whereas adult television advertisements from these QSR companies emphasized the taste, portion size and price of food products, children’s advertisements emphasized toy premiums and movie tie-ins, brands and logos. Children’s advertisements also emphasized the street view of the restaurant, which may help children to recognize it as they drive by with their parents. The clear emphasis in child QSR advertisements on toy premiums and movie tie-ins suggests that during the study period, CARU and CFBAI self-regulatory pledges were associated with little advertising emphasis on actual food products sold to children. Moreover, the children’s advertisements emphasized techniques that the companies’ self-regulatory body has identified as potentially misleading. Our findings are consistent with the experience of Australia, where food companies have failed to live up to self-regulatory standards there [22].

Given health concerns about obesity and its relation to fast food consumption, enhanced oversight of QSR marketing to children at the local, state and federal level is needed to align QSR advertising to children with health promotion efforts and existing principles of honest and fair marketing to children. Forcing accountability through periodic evaluation of food industry advertising, [23] as we have tried to exemplify here, is one basis for successful self-regulation. We suggest that annual evaluations are needed. In order to be effective, however, the monitoring needs to be conducted by an agency like the FTC. If the same problems continue to be found in more contemporary advertisements despite continued self-regulation, further governmental action aimed at children’s food advertising may be warranted.

Our finding of the lack of substantial emphasis on food in the self-regulatory environment is limited by the sample frame, which ended in June 2010, and we cannot comment on the content of children’s advertisements since then. Also, our content analysis involves adults observations about advertisements aimed at children. Future research should be done to test whether children tend to focus on premiums and tie-ins in QSR television advertising. Our decision to focus only on ad content is not able to address whether certain ads were targeted to low SES or minority groups, those for whom obesity is most prevalent. Future studies should address whether food ad targeting enhances such health disparities. Finally, our study does not assess whether seeing children’s advertisements prompts requests for toys or visits to fast food restaurants, or whether such exposure is related to obesity. These areas seem fertile ground for further research.

In summary, this study examined children’s QSR food advertisements that aired nationally between 2009 and 2010, a period when both McDonalds and Burger King aired 99% of QSR advertisements aimed at children, promised to emphasize healthy food, and to de-emphasize toy premiums and movie tie-ins. Although some of the foods presented in children’s meals could be characterized as “healthy,” little emphasis was placed on actually showing the food compared with adult advertisements from the same companies, and toy premiums and tie-ins were presented prominently in both the visual and audio elements of these advertisements. We conclude that these companies did not follow through with their self-regulatory promises during the study period.
